# Editorial: Neural and Computational Modeling of Movement Control

**DOI:** 10.3389/fncom.2016.00090

**Published:** 2016-08-31

**Authors:** Ning Lan, Vincent C. K. Cheung, Simon C. Gandevia

**Affiliations:** ^1^School of Biomedical Engineering, Shanghai Jiao Tong UniversityShanghai, China; ^2^Division of Biokinesiology and Physical Therapy, University of Southern CaliforniaLos Angeles, CA, USA; ^3^School of Biomedical Sciences, The Chinese University of Hong KongHong Kong, China; ^4^Neuroscience Research AustraliaSydney, NSW, Australia

**Keywords:** neural circuits, computational modeling, sensorimotor control, movements, postures

## Introduction

There exists a gap from experimental data to the understanding of neural control of movements. This research topic was dedicated to promote computational modeling approach that can facilitate data interpretation (Niu et al.; Ranjbaran and Galiana; Pearson et al.; Sharif Razavian et al.; Malik et al.), elucidate control theories (Ueyama; Ota et al.; Takemura et al.), shed light on systemic mechanisms (Buhrmann and DiPaolo; Pearson et al.; Li et al.), suggest testable hypothesis (Loeb and Tsianos; Jiang et al.), and aid design of rehabilitation or therapeutic strategies (Zitella et al.). The 14 articles reflected these different aspects of computational modeling in bridging this gap between functions of neural circuits and observable behaviors. This research topic demonstrated that computational modeling is playing a more and more prominent role in sensorimotor control studies.

## Editorial

Our knowledge of the neural mechanisms of movement generation is mostly derived from experimental data obtained in animals and humans. For a more comprehensive and holistic understanding of motor control, the ever-mounting experimental information must be integrated to allow general principles of sensorimotor control to emerge. Progress in this integration has been stagnant owing to the fragmented nature of many available data sets, usually recorded from constrained preparations or under very specific behavioral conditions. One mathematical framework for consolidating data is to fit the data using mathematical equations with optimized, “best-fit” parameters (e.g., choosing parameters that maximize the data variance accounted for by the equations). This approach has evolved from a “black-box” type of modeling to building biologically and neurophysiologically realistic, multi-scale models. The success of the latter approach hinges on the assumption that the models represent the underlying computations of neural signal processing in central sensorimotor system.

The modeling approach advocated here is knowledge-based deduction with simulations using computational models. Simulations must be compared to observable states of the system to validate the hypothesis advanced (Cordo et al., 2002; Stein et al., 2004; Lan and He), or to propose new testable hypotheses (Bullock, 1993). Building a multi-scale model of the sensorimotor system from muscles, proprioceptors to skeletal joints, spinal regulating centers, and central control circuits exemplifies part of this endeavor (Cheng et al., 2000; Lan et al., 2005; Mileusnic et al., 2006; Alstermark et al., 2007; Song et al., 2008a,b; Hao et al., 2013; He et al., 2013). The review article by Loeb and Tsianos outlined the necessary elements and challenges in this approach, which is the first step toward integrating a vast body of experimental data into a general mathematical framework for simulation.

Experimental mapping of neural circuits, or neural modeling, provides the essential foundation upon which mathematical descriptions of the neural system are formulated (Baldissera et al., 2011; Prochazka and Ellaway, 2012). New technologies, such as optogenetics (Bernstein and Boyden, 2011; Fenno et al., 2011), have added out ability to dissect neural circuits in the brain and spinal cord. Alstermark and Ekerot described their work in identifying the spino-cerebellar closed-loop circuit via the brainstem lateral reticular nucleus. Jiang et al. reviewed the anatomy and physiology of the direct and indirect spino-cerebellar tracts and illustrated how these pathways, originating in the spinal cord, may be the neural substrates for the transmission of internal feedback signals in control models. They proposed a new, testable hypothesis, that the direct pathway is primarily involved in rhythmic motor acts such as locomotion, while the indirect pathway provides the neural substrates for pre-cerebellar sensorimotor integration required for dexterous limb movement.

The complexity of a model depends on the specific question one wishes the model to address. Niu et al. developed a hardware model of a spinal reflex that demonstrated real-time capability in simulation. Ranjbaran and Galiana used a context-dependent model to shed light on potential underlying neural mechanisms of the vestibulo-ocular reflex. Pearson et al. created a four-link biomechanical model of a cat hind leg to determine how mechanical and neural factors contribute to the updating of working memory of barrier location during locomotion. Sharif Razavian et al. developed an alternative method to understand muscle synergy by using a biomechanical model that is associated with an optimal solution for task control. Malik et al. constructed a bioinformatic model that incorporated limb biomechanics embedded with six muscle spindles to predict sensory outputs.

Behaviors are often the outcome of a complicated process of neural computations in the brain and spinal cord (Shadmehr and Wise, 2005). Computational models can be of much help in elucidating the roles of individual neural computations in movements. Buhrmann and DiPaolo used a simple two-link model to examine whether peripheral feedback is sufficient to coordinate multi-joint motion—i.e., the motion in the presence of intersegmental interaction torques. Their simulation showed that it is plausible that spinal circuity can control multi-joint movements even in the absence of internal models of intersegmental dynamics or learned compensatory motor signals. Ranjbaran and Galiana presented a hybrid nonlinear bilateral model for the horizontal angular vestibulo-ocular reflex (AVOR), and investigated a viable switching strategy for the timing of nystagmus. Simulation results replicated experimental data well in all conditions. Li et al. used a corticospinal, virtual-arm model to investigate the central coordination of alpha and gamma controls to muscles and their muscle spindles for movement generation. Simulation results indicated that simple patterns of alpha and gamma drives are sufficient to control a range of movements, and that propriospinal neurons (Alstermark et al., 2007; Hao et al., 2013) may play an essential role in pre-motor processing of descending commands for movements.

It has long been presumed in motor neuroscience that movement generation begins with a motor planning, followed by a motor execution (Hogan, 1988). How motor planning and execution are accomplished has been subjected to much theorizing (Ajemian and Hogan, 2010). Computational models have been used to address these theoretical questions. Ueyama proposed a new control scheme, called mini-max feedback control, in which motor commands are generated by minimizing the maximal cost to the action resulting from worst-case uncertainty; this scheme outperformed the popular optimal feedback control scheme (Todorov and Jordan, 2002) both in stability and task-goal achievement. Ota et al. also studied the question of motor optimality. They argued that human motor planning is suboptimal when the gain associated with the action is “asymmetric.” Takemura et al. followed up on the question of motor planning in light of uncertainty by studying human reach-to-grasp task when the target was visually occluded, a condition that led to a larger peak grip aperture when compared with conditions with vision. To account for the increased grip aperture, they formulated a model based on the assumption that grip aperture is controlled to compensate for motor variability and sensory uncertainty.

An important motivator of computational modeling is the potential use of this body of knowledge to design new, efficacious interventions for treating movement disorders (Reinkensmeyer et al., 2016). This use of computational models is exemplified in the article by Zitella et al. They employed a computational model to evaluate the therapeutic potential and side effects of deep brain stimulation (DBS) of the pedunculopontine tegmental nucleus (PPTg) in a Parkinsonian monkey. This model predicted how different DBS stimulation parameters produced different activations of the nerve fibers surrounding the PPTg.

## Conclusions

This research topic demonstrated that computational modeling is playing a more and more prominent role in the studies of postural and movement control. With increasing ability to gather data from all levels of the neuronal sensorimotor system, there is a compelling need for novel, creative modeling of new and existing data sets, because the more systematic means to extract knowledge and insights about neural computations from these data is through computational modeling. While models should be based on experimental data and validated with experimental evidence (Ajemian and Hogan, 2010), they should also be flexible to provide a conceptual framework for unifying diverse data sets, to generate new insights of neural mechanisms, to integrate new data sets into the general framework, to validate or refute hypotheses and to suggest new testable hypotheses for future experimental investigation (Bullock, 1993). It is thus expected that neural and computational modeling of the sensorimotor system should create new opportunities for experimentalists and modelers to collaborate in a joint endeavor to advance our understanding of the neural mechanisms for postural and movement control.

## Author contributions

NL and VC drafted and edited the final version of the editorial. SG edited the final version.

## Funding

NL is funded by The Natural Science Foundation of China (No. 81271684 and No. 61361160415) and The Ministry of Science and Technology of China (No. 2011CB013304). VC is supported by startup funds from The Chinese University of Hong Kong.

### Conflict of interest statement

The authors declare that the research was conducted in the absence of any commercial or financial relationships that could be construed as a potential conflict of interest.
